# Femoral Aneurism

**Published:** 1849-09

**Authors:** 


*Femoral Aneurism.*—Mr. Tufnell communicated to the Surgical Society of Ireland (Feb. 17, 1849) the following extremely interesting case of femoral aneurism. The subject of it was an Irishman, a sergeant of infantry, aged 34, five feet ten inches in height, a strong and very healthy man, of temperate habits, and extreme moral courage. On the 10th of March, 1848, whilst at drill, performing the extension motions, and endeavoring to touch the ground without bending the knees, he felt something snap in the fold of the left groin. He did not suffer pain or inconvenience, and made no report of the circumstance. The day but one following he marched a distance of eighteen Irish miles. His foot and leg swelled upon this occasion, and continued to do so for several days, when, finding the edema so great as to prevent him from wearing his boot, he went into hospital, complaining of being footsore from the march. He rested in bed for a short time, and the swelling gradually subsided. He returned, therefore, to his duty, and continued at it for upwards of a week, when, being engaged in the escort of provisions, and finding himself unequal for the duty, he was readmitted into the hospital, complaining of edema and leg weariness as before. Rest in the recumbent position again relieved the swelling, and he reported himself as fit for his duty. He was accordingly a second time discharged, and was immediately sent (being a steady, intelligent man) on the recruiting service to England.

Here, during a period of several months, he performed every duty required of him. He stated that, shortly after leaving the regiment, the swelling of the leg became constant, and a small tumour gradually formed in the fold of the left thigh, which at first he could perceive in the night, when in bed, ticking like a clock, but that afterwards, as the swelling increased, the pulsation became indistinct. He never sought for medical relief, but was daily walking about, searching for recruits. This state of things continued until early in the month of October, when the pain in the thigh and leg (especially after stand ing for several hours), became so severe as to oblige him to apply for permission to return to his regiment. He was accordingly recalled, but on rejoining his corps, he made no further complaint, and continued to perform the ordinary duties of a sergeant until the 22d of December last. On that day he went to the hospital, and exhibited to the medical officer of his corps a large, pale tumour, situated at the fold of the left thigh, immediately below Poupart's ligament, and encroaching upwards upon the abdomen.

The emergency of the case was reported by the medical officer to Sir James Pitcairn, the inspector-general of hospitals, who immediately ordered a medical board to assemble and report upon the practicability of removing the man to Dublin, in order that he might have the advantage of treatment in the Royal Infirmary. The board having reported that he could be safely removed by short stages and under proper escort, he was accordingly ordered up, and arrived at the Military Hospital on the 20th of January, and was placed under Dr. Fox's care.

The aspect of the tumour at this time was frightful from its size, and bore somewhat the appearance of fungous hæmatodes. It rose in a sugarloaf form from the centre of the left groin, measuring one foot eleven inches round the base, and eleven inches across the top. Its base was pink and slightly inflamed, its apex purple, with the skin considerably abraded, marking an incipient slough. Pulsation was visible by placing a piece of paper on the surface, or applying the hands to the sides of the tumour, whilst a distinct single bruit could be heard throughout. The tumour encroached greatly upon the abdomen, its upper border being level with the brim of the pelvis. The thigh was enormously swollen, being twice the size of the other; but the limb below was not correspondingly increased, the foot being only one inch and a half, and the calf of the leg only three inches larger than that of the opposite side. The temperature was equal in both; the posterior tibial artery could not be felt on the affected side, but pulsation in the popliteal was doubtful.

To view this man lying in bed, no suspicion of such disease could have been entertained. His eye was clear and bright, his countenance placid, not in any way expressive of suffering, and he made no complaint. When questioned, he said that the pain, which had been severe during the journey, had subsided, and that he felt tolerably well. His tongue was moist; his pulse one hundred, regular and soft; he had slept soundly the preceding night under the influence of an anodyne, and awoke free from headache; all that he complained of was, that his appetite was failing, and that he felt correspondingly weak.

A consultation on the case held on the 22d, Sir James Pitcairn, Staff-Surgeons Sinclair, Scott, and Fox, and Assistant-Surgeons Barrett and Ivey, with Sir Philip Crampton, Mr. Cusack, and Dr. Adams being present. It was unanimously determined, under the circumstances, not to interfere, unless compelled to do so by the sack giving way, but to leave the case to nature, in the hope that, as the pulsation was very indistinct and gradually declining, it might spontaneously cease, the artery be obliterated above, the sack become filled with coagula, and the aneurism eventually slough out; such a termination having twice occurred in somewhat similar cases in the practice of Sir Philip Crampton.

Cold lotions were ordered to be applied to the tumour, the patient's strength to be kept up by strong beef-tea, and rest to be procured by opium at night.

On the 23d, no change whatever had taken place.

On the 24th, he was not so well. He had perspired profusely through the night, the sweat having an acid, rheumatic odor. The tongue was dry, and he complained of thirst. The pulse was increased in frequency, being one hundred and twenty in the minute. Pain had returned in the tumour, which, he said, felt as if hot water were poured upon it. The pulsation was not perceptible and the bruit could be distinguished only by applying the ear directly to the tumour: it was inaudible through the medium of a stethoscope. The surface of the tumour was more livid, and edema slightly increased; but the temperature of the leg was natural and equal to that of the opposite limb.

The same treatment was ordered to be continued.

On the 25th there was an apparent improvement in his general condition, the pulse having come down to one hundred and eight, the tongue become moist, and the thirst less urgent. The pain in the tumour had subsided, and he complained only when the limb was stirred; but the tumour itself had increased in size, its surface being livid, indeed almost black, the skin abraded, and weeping sanguinous fluid sufficient to tinge the cloth by which the lotion was applied. The discoloration of the surface extended over the brim of the pelvis, giving an ecchymosed appearance to the integuments down as low as the side of the nates. The temperature of the limb was unchanged.

During the course of this day, a bulging prominence presented itself to the outer edge of the tumour, having much the appearance of a knuckle of livid intestine.—Drs. Fox and Barrett, who were in close attendance upon the patient, now determined to cut down through the centre of the tumour, and tie the vessel as it entered the sac, as soon as ever the first oozing of blood should appear.

Their opinion, indeed, was not to wait for the occurrence of this event, lest the jet of blood should be too great for the patient to bear, but to anticipate what now appeared to be almost inevitable. The general impression, however, upon the minds of the members of the consultation being, that, from the mass of coagulum then lying between the artery and the surface of the tumour, the vent would not be followed by violent hemorrhage, they were inclined to hold their hands, and give nature every chance, waiting ready to interfere as soon as all further hope was removed.

This event occurred at 6 P. M. The patient was in the act of moving the limb when the aneurism suddenly burst—the blood spouting in a heavy jet full three feet from the bed! Compression upon the aorta was instantly made by Dr. Barrett, whilst Dr. Fox, assisted by Dr. Ivey, carried a free incision through the centre of the tumour, (turning out the mass of clot and gore contained in the sac,) and quickly placing a ligature round the mouth of the artery as it entered the sac. No hemorrhage took place, and not an ounce of blood was lost during the operation, which was borne with the greatest fortitude by the patient, who expressed himself as relieved by the removal of the tension of the tumour. An opiate was subsequently administered, and he slept soundly for four hours. He then dozed at intervals, between which he appeared to become fainter and fainter. Wine had been freely given both before and after the operation; but, in spite of all stimulants, his extremities gradually grew cold, and he died slowly off, at four o'clock, on the morning of the 26th, ten hours after the vessel had been secured. There was no return of hemorrhage from the lower end of the artery, and the ligature after death was found firmly and closely fixed round the mouth of the vessel. And here I cannot refrain from testifying to the able manner in which this operation was performed, under circumstances of no ordinary difficulty, by Dr. Fox and the gentlemen associated with him. The sudden loss of blood, consequent upon the rupture of the sac, though borne against for a time, appears to have been too great for the powers of life ultimately to withstand.

Upon examining the body thirty hours after death, it presented a very different appearance, as far as regards the tumour, from what it bore during life, its contents being removed, and the integuments of the groin lying flaccid and flat. The superior extremities were muscular, and the thorax and ribs well clothed, although, judging from the comparative size of the lower extremities, as exhibited in the drawing and cast, it might be inferred that the patient was emaciated, and ran down. On opening the cavity of the abdomen, the viscera and peritoneum were in a perfectly healthy state. The iliac arteries were healthy, and no abnormal condition of the parts could be detected, except that the tendons of the oblique and transversalis muscles on the left side were loose and lying in a fold, having, during life, been distended and pushed upwards by the aneurismal sac. The lymphatic glands were numerous and enlarged.

Upon dissecting the aneurism itself by reflecting the integuments, the sac was found to originate from the femoral artery, immediately after passing from beneath Poupart's ligament. The sac was of an hourglass form, being constricted by the fascia lata, the portion beneath the fascia extending in an oblique line downwards to the canal in the adductors ; the portion above the fascia rising directly forwards and upwards, forming the tumour, represented by the cast. Anteriorly, the tumour was covered by the sartorious muscle, which was spread over it to the width of four inches ; posteriorly, it sank deep into the structures of the thigh, pressing against the capsule of the hip-joint, as well as against the neck of the femur. This pressure had been sufficient to create inflammation of the synovial membrane of the joint, as well as the cartilage on the head of the bone, which bore an arborescent appearance from the red vessels ramifying in it. The periosteum was also removed from the anterior part of the neck of the femur, the bone being abraded and rough. To the outer side the sack was bounded by the shaft of the femur, and to the inner side it wended its way through the adductor muscles, the fibres of which, (together with all the others in the neighborhood), were stretched around the sac, forming as it were its superficial coat, the middle being a condensation of the tissues, and the innermost a lining of fibrine. The sac was still circumscribed below.

Upon examining the preparation, and tracing the iliac artery from above, it appears to be perfectly healthy, and of a natural size. It enters the sac by a small rounded opening, the edges of which are abraded by the ligature which was placed upon it as a temporary restraint to the hemorrhage, prior to securing the vessel above. The sac itself is polished throughout, lined by a thin layer of fibrine, rendered smooth by the friction of the blood. It measures, in its present state, after being immersed in alum, (and thereby greatly constringed,) eleven inches in length, and seventeen in circumference.

Its inferior connection with the artery is similar to the superior, being perfectly round and even, situated from it at a distance of three inches in a direct line. The femoral artery beyond the sac, is perfectly healthy, running in its usual course to the canal in the adductors, at which spot it has been divided across. The artery, therefore, retains its normal condition, except as regards the formation of the aneurismal sac.

Not so the vein. Viewed from above, it is seen to be separated from the artery, the sac of the aneurism carrying it off at an angle, and pushing it rather to the outer side of the artery, and after traversing the superior surface of the aneurism for a couple of inches, it becomes obliterated, not permitting even a probe to pass. Below the tumour it has become completely transposed, lying altogether to the outer side of the artery, and maintaining this situation until it reaches the canal in the adductors, where it winds round the vessel and regains its natural position. From the sac, for the distance of two and a half inches, it has become obliterated, assuming the appearance of, and feeling to the touch, like an artery plugged up with fibrine. Below this point it becomes pervious, and at the entrance to the tendinous canal resumes its normal size and shape. The vessels arising from the femoral artery are all obliterated at the sac, and, as it were, imbedded in its walls. The crural nerves are greatly stretched, flattened, and apparently enlarged.

These are the principal points to be observed in the morbid specimen itself. "The one, however," remarked Mr. Tuffnell, "to which our attention as surgeons should be directed, is: What mode of treatment could have been adopted that would have afforded any prospect of saving life?

"In the first place, the tumour had acquired such an enormous size, and so encroached upon the contents of the abdomen, lying, as it were, over and upon the external iliac artery, as to render it almost impossible to get at this vessel while the sac remained entire; the incision ordinarily made for the purpose of securing the external iliac artery, instead of coming down upon it, would have disclosed only the sac itself, with the external oblique and transversalis fascia distended upon it. The projection of the tumour into the abdomen precluded, therefore, this operation, unless it was preceded by evacuation of the contents of the sac, thereby restoring Poupart's ligament and its component tendons to their usual site. This, then, I consider, (if a similar case should occur,) ought to be the primary step, and that the operation performed by Dr. Fox, of cutting through the centre of the tumour, turning out its contents, and tying the vessel as it entered the sac, should be the first thing done. The next step should be the placing of a ligature on the external iliac artery; and the final one, the removal of the limb at the hip.

"'Heroic' as this may appear, I feel certain that nothing short of it could offer any chance of life. Suppose that, instead of resorting to removal of the limb, we secure the artery only. We leave the patient then with the supply of blood to the limb almost totally cut off, for the pressure of the sac has obliterated nearly every branch of communication with the vessels above, and gangrene of the limb, I may say, would be inevitable. Should this event, by some possibility, not occur, there would still remain a cavity in the muscles of the limb, a vast suppurating sac, from which hectic would assuredly follow, and quickly run the patient down.

"There would also, in addition, exist general synovitis of the joint and erosion of the neck of the bone. These, however, depending upon a cause—namely, the pressure of the sac—which would now be removed, I will not throw much stress upon them, as they might spontaneously cease, and the reasons I have given above are alone, I think, fully sufficient to warrant the operation of removing the joint of the hip.

"This form of amputation has for the most part hitherto proved fatal, not from the loss of blood, but from the shock to the nervous system in general, and it remains for experience to show whether, under the influence of chloroform, we may not anticipate happier results.

"Such, I consider, will be the case; for, judging from the numerous instances in which I have myself used and seen chloroform employed by others, there has not appeared to be greater appreciation of injury to the frame where amputation has been resorted to than where a single tooth has been removed."

"Dr. Bellingham had had the opportunity of seeing this case, and would take the liberty of offering a few remarks upon it. Many similar cases might, he observed, be found in the works of the older writers on surgery, in some of which a spontaneous cure took place, in consequence of the aneurism increasing so much in bulk as to press upon and obliterate the artery above the seat of the aneurism, after which suppuration ensued in the sac, and the patient ultimately recovered. In the case before the society, when the patient entered the Military Infirmary, there appeared to be no chance of a spontaneous cure taking place, the skin being, at that time, on the point of giving way. If, on the other hand, the common iliac artery had been tied, gangrene would undoubtedly have set in, or the sac would have suppurated, and the patient have been finally run down by hectic fever. With respect to Mr. Tufnell's suggestion of amputation at the hip joint, he was inclined to think that an operation which had succeeded in so few cases must inevitably have proved fatal in a case of so serious a nature as this, where the limb was extensively engorged. He considered that the plan recommended by Mr. Adams was that which gave the only chance of success. Whether the disease was left to itself, or whether the external iliac artery was tied, he must equally have died; but he thought the sac (as Mr. Adams had recommended), might with advantage have been laid open, the coagula turned out, and the case treated as a wounded artery, the vessel being tied both above and below the aneurismal sac."—*Dublin Medical Press.*

